# Contraceptive use and method preference among HIV positive women in Addis Ababa, Ethiopia: a cross sectional survey

**DOI:** 10.1186/1471-2458-14-566

**Published:** 2014-06-06

**Authors:** Hussen Mekonnen Asfaw, Fikre Enquselassie Gashe

**Affiliations:** 1 Department of preventive medicine, School of Public Health, Addis Ababa University, Addis Ababa, Ethiopia

**Keywords:** Contraceptive use, HIV positive women, HAART use, Addis Ababa, Ethiopia

## Abstract

**Background:**

Prevention of unplanned pregnancies among people living with HIV is essential component of “Global Plan” even in the context of expanded access to highly active antiretroviral therapy (HAART). The study aimed to assess whether contraceptive use and method preference varied by the use of HAART among HIV positive women in Addis Ababa, Ethiopia.

**Methods:**

A cross sectional facility based survey was conducted from June to October, 2012 information was gathered using interviewer administered questionnaire and document review was conducted to confirm HIV status and clinical review. A sample of 1418 HIV positive women including 770 women receiving HAART and 648 HAART-naïve recruited randomly from different health institutions in Addis Ababa. Data were principally analyzed using logistic regression.

**Result:**

Overall, 71% women reported using contraception (75% among HAART users and 65% HAART naïve women). Male condom and injectables are the most preferred contraceptive methods among both groups. The odds of contraceptive use among HAART users was higher (AOR 1.60, 95% CI; 1.30-2.12) than HAART naïve women. In addition to this, presence of partner (AOR 2.32, 95% CI 1.60-3.40), disclosure of HIV status to husband (AOR 2.23; 95% CI 1.21-4.12), presence of living children: one (AOR 1.7; 95% CI 1.03-2.40), two (AOR 2.6; 95% CI 1.7-4.02) and three (AOR 3.3; 95% CI 1.90-5.60) respectively were found to be predictors of contraceptive use among HIV positive women.

**Conclusion:**

The contraceptive profile of women in the study area mainly dependent on male condom use, this indicates the need to better integrate tailored counseling and contraceptive options with care and support activities that targets HIV positive women. Moreover, emphasis should be given to dual contraceptive method use along with their regular follow up irrespective of their HAART use.

## Background

Sub-Saharan Africa is the region most severely affected by HIV [1]. Women of productive age account for 58% of the people living with HIV [2] and 53% of all adult deaths [3]. In Ethiopia, more women (2.9%) than men (1.9%) are living with HIV [4]. Most of these women are particularly vulnerable to HIV due to complex burden they have [5] including physiological, social vulnerability and gender inequalities [3].

Furthermore, HIV positive women have risk of unplanned pregnancies [6]. Worldwide, two in every five pregnancies are unplanned [7]. Previous studies of 42 sub-Saharan Africa countries, 10-65% of women reported that their last pregnancy had been unplanned [8]. Similarly, 62% of women on ART from South African [9] and 59% of HIV positive women from Kenya reported unplanned pregnancy [10].

Prevention of unintended pregnancy among HIV positive women is the second element of prevention of mother to child transmission of HIV (PMTCT) [11] which is an important means to address the associated problems of HIV positive women and children [12]. It is reported to be cost effective [13], improves quality of life [14], reduces maternal and child mortality [3,12,15-17] and reduces number of positive infants by keeping their mother alive [18].

Significant improvements have been demonstrated by PMTCT in low and middle income countries [19]. The percentage of pregnant women with HIV receiving antiretroviral therapy(ART) using PMTCT in sub Saharan Africa increased to 50% [3]. However, PMTCT efforts to date gave less emphasis to the contraceptives options [20].

Despite the potential contribution of family planning(FP) to the prevention of HIV infection [12] and unintended pregnancies [3], contraceptive use in sub-Saharan Africa remains low [21]. The percentage of married women aged 15–49 using modern contraceptive methods ranges from 12% in Mozambique and 14% in Ethiopia to 27% in Rwanda [4]. In addition, previous reports indicated that twenty five percent of women with HIV worldwide and one in four married women from sub-Saharan Africa has unmet need for family planning [22].

Even in the context of expanded access to ART for HIV-infected pregnant women, family planning still needs to be strengthened to avoid unintended pregnancies [23]. Existing evidence revealed that women living with HIV including those taking ARVs can use almost all contraceptive methods safely and effectively [15,16] with variation in method preference [5,12,24-27].

Previous studies from different parts of Ethiopia reported that of those who have ever used at least one contraceptive method after HIV diagnosis, condom, injectables and abstinence were reported to be the most preferred methods [28-30]. However, information on informed choice of contraceptive methods, reasons for selection of methods and the influence of HAART on the contraceptive use of women entering HIV care is scarce.

Ethiopia is the second populous country in Africa [31], characterized by high fertility of 5.4% children per woman [4], having more children is highly valued [32]. Moreover, majority of women deliver at home (usually without skilled attendant) where complications of childbirth cannot be addressed, including the risk of mother-to-child transmission of HIV [31]; identification of factors associated with contraceptive use and method preference among HAART using women is an important issue to be addressed.

Thus, given the importance of contraceptive use in preventing unintended pregnancy, perinatal transmission of HIV [3] and ART scale up in Ethiopia; it is worth to assess contraceptive use and method preference of HIV positive women. It is anticipated that this research will update existing knowledge, inform policy makers and programmers to support safer and healthier reproductive options among HIV positive women in the study area.

## Methods

The study was conducted in health facilities of Addis Ababa, Ethiopia. In the country, Antiretroviral treatment began in 2003 and free ART was launched in 2005 [33]. Since then, a total of five public hospitals and twenty five health centers have been offering ART [34]. About 124,983 people living with HIV were enrolled of whom 76,035 have started and 54,667 are currently on ART in the city [4]. Amhara is the predominant ethnic group and Amharic is commonly spoken language in the study area [35].

### Study design and sampling procedures

A cross sectional survey was carried out at selected public health facilities (hospitals & health center) of Addis Ababa between June and October, 2012. Non-pregnant women aged 18–49 years, reported having sexual relationship six months prior to the data collection period were eligible to participate in this study.

Sample size was computed using two-proportion formula with the assumption of proportion of contraceptive use among HIV positive women receiving HAART and HAART naïve being 86% and 82%, respectively [12], with a minimum detectable odds ratio of 2 at 5% level of significance power of 90% with a design effect of 2 and 5% addition for non-response rate a total sample size of 1924. However, the number of women who satisfy the eligibility criteria at the records of the health facilities was 1418 (770 HAART users and 648 HAART- naïve).

Study participants were selected using multi-stage sampling technique. Initially, five hospitals and seven health centers were selected by lottery methods, and then the total sample size was proportionally allocated to the selected facilities according to the client load in each institution. Finally individual study subjects were selected using a systematic random sampling technique from the records of each selected facility.

### Data collection

Data were collected using interviewer administered structured questionnaires adopted from previous similar study [12]. The questionnaire has been translated in to the local language (*Amharic*) by experts in both languages and back translated to English by another person to ensure consistency and accuracy. Data collectors were all female nurses recruited based on their previous experience in data collection, those who had previous training on ART and/or HIV counseling and fluency of the local language. Moreover, training was given for five consecutive days on interview techniques, sampling and ethical issues, emphasizing the importance of safety of participants and interviewers, minimization of under reporting and maintaining confidentiality. A pre-test of the questionnaire was conducted in selected ART units which were not included in the main study. The data collection process was closely supervised by the principal investigator.

### Measurements

The primary outcome variable was self-reported contraceptive use for the previous six months prior to the data collection period. In this study contraceptive use was defined the use of any method including injectables (Depot), implants (implanol or jadelle), oral contraceptive pill (OCP), intra uterine contraceptive device (lUCD), male/female condom, diaphragm, permanent methods such as tubal ligation and hysterectomy. Dual contraceptive method was defined as use of primarily the male condom with hormonal or other contraceptive methods like IUCD. Consistent condom use was defined as the use of condoms always or frequently in all vaginal sexual relationships with casual and/or steady partners. Women who used condoms sometimes or rarely were regarded as inconsistent condom users.

Independent variables in this study included were age, education, employment, monthly household income, sexual partnership status, HIV sero-status of partner/husband, number of living children, HIV test of children, disclosure of HIV status to partner/husband and discussion with partner/husband or health workers about contraception. Medical record review was also conducted to confirm HAART history and obtain clinical data including World Health Organization stage of disease and CD4 cell count.

Antiretroviral therapy (ART) is treatment of people infected with human immunodeficiency virus (HIV) using anti-HIV drugs [36]. The standard treatment consists of a combination of at least three drugs (often called “highly active antiretroviral therapy” or HAART) that suppress HIV replication [37]. Highly active anti retroviral therapy (HAART) use defined as use of one of three antiretroviral medications either Efaveranze (EFV) or Neverapin (NVP) based [5,12,38] first line drugs or use of combination drugs with protease inhibitors(PIs) backbone of second line drugs [38]. We considered women to be HAART users if they had been receiving HAART for at least one month and HAART-naïve if they had never taken HAART, prior to the data collection period.

### Analysis

The pre coded responses were double entered in EPI Info version 3.5.2 software, for checking its consistency then was exported to SPSS for window version 20 for statistical analysis. Uses, preference and reasons for selection of contraceptive methods are presented using descriptive statistics. Variables found to be significant at bivariate level, (P < 0.05), were selected and included in to multiple logistic regression models. Then multiple logistic regression analyses were used to calculate Odds ratio with 95% confidence interval to estimate association and to control the potential confounding variables. Strength and direction of the association between contraceptive use and potential socio-demographic, reproductive health and clinical factors were presented using odds ratios relative to the reference category and 95% confidence levels.

### Ethical considerations

The research was approved for scientific and ethical integrity by the Institutional Review Board (IRB) of College of Health Sciences, Addis Ababa University. Written permission was obtained from health bureau of the Addis Ababa city government. Consent was obtained from medical directors and respective unit heads at each health institutions. Verbal consent was also obtained from individual clients. In order to make informed decision sufficient information was given to each participant. Confidentiality was strictly maintained for each piece of information and the interview was conducted in strict private place. At the end of the interview general information, referral and follow up linkages were made for those who need.

## Results

### Socio-demographic characteristics of study participants

Of the total 1418 women who participated in this study, 423 (29.8%) were in the age range of 30–34 years, with mean (standard deviation) of 31.1(±5.5). The study participants were predominantly Orthodox Christians 1109(78.2%) and Amhara 849(59.9%) by religion and ethnicity, respectively. Occupationally, 373 (26.3%) participants had their own private business and 292(20.6%) were housewives. Concerning educational status of the participants, 538 (37.9%) have completed grade 1–8, 466(32.9) grade 9–12 and 133(9.4%) with higher education. Four hundred nineteen (29.5) earn monthly income of less than 1000 Ethiopian birr and 390 (27.5%) women were with no monthly income. Concerning marital status, about 62% of the participants are currently married/cohabited and 242(17.1%) were single (Table 1).

**Table 1:** Socio-demographic characteristics of HIV positive women in Addis Ababa, Ethiopia (n = 1418)

**Socio-demographic characteristics**	**HAART users (n = 770)**	**HAART Naïve (n = 648)**	**Total**
	**No (%)**	**No (%)**	**No (%)**
**Age (in years)**			
18-24	58 (7.5)	91 (14.0)	149 (10.5)
25-29	219 (28.4)	20 3(31.3)	422 (29.8)
30-34	245 (31.8)	178 (27.5)	423 (29.8)
35-39	186 (24.2)	122 (18.8)	308 (21.7)
40-49	62 (8.1)	54 (8.3)	116 (8.2)
Mean (SD) Age in years	31.6 (±5.2)	30.4 (±5.8)	31.1 (±5.5)
**Ethnicity**			
Amhara	462 (60.0)	387 (59.7)	849 (59.9)
Oromo	167 (21.7)	147 (22.7)	314 (22.1)
Gurage	70 (9.1)	54 (8.3)	124 (8.7)
Tigrea	47 (6.1)	32 (4.9)	79 (5.6)
Others (Silte,gamo, welayta, yem, Sidama)	24 (3.1)	28 (4.3)	52 (3.7)
**Religion**			
Orthodox Christian	604 (78.4)	505 (78.0)	1109 (78.2)
Muslim	84 (10.9)	76 (11.7)	160 (11.3)
Others (Protestant, catholic, Jubbah)	82 (10.6)	67 (10.3)	149 (10.5)
**Marital status**			
Single	126 (16.4)	116 (17.9)	242 (17.1)
Married/cohabited	488 (63.4)	397 (61.3)	885 (62.4)
Widowed	81 (10.5)	55 (8.5)	136 (9.6)
Divorced	75 (9.7)	80 (12.3)	155 (10.9)
**Educational status**			
illiterate	91 (11.8)	78 (12.0)	169 (11.9)
Informal education	50 (6.5)	62 (9.6)	112 (7.9)
Grade 1–8 completed	273 (35.5)	265 (40.9)	538 (37.9)
Grade 9–12 completed	290 (37.6)	176 (27.2)	466 (32.9)
above 12 grade	66 (8.6)	67 (10.3)	133 (9.4)
**Occupation**			
unemployed	131 (17.0)	133 (20.5)	264 (18.6)
Housewife	166 (21.6)	126 (19.4)	292 (20.6)
Daily laborer	96 (12.5)	112 (17.3)	208 (14.7)
Merchant	40 (5.2)	36 (5.6)	76 (5.4)
CSW	18 (2.3)	13 (2.0)	31 (2.2)
Government worker	90 (11.7)	84 (30.6)	174 (12.3)
Private business	229 (29.7)	144 (22.2)	373 (26.3)
Monthly Income (in Birr)*			
No Income	192 (24.9)	198 (30.6)	390 (27.5)
Birr below 500	217 (28.2)	144 (22.2)	361 (25.5)
Birr 500-1000	227 (29.5)	192 (29.6)	419 (29.5)
Birr 1001-3000	112 (14.5)	97 (15.0)	209 (14.7)
Birr above 300	22 (2.9)	17 (2.6)	39 (2.8)

*Birr = (1USD = 18.80 ETB).

### Reproductive and clinical characteristics of study participants

About 55%(422/769) of HAART users and 45% (347/769)HAART naïve women had recent CD4 counts of 350 cell/mm3 and 42% (363/863) and 58% (500/863) HAART users and HAART naïve, respectively were in WHO stage of disease I or II. About 70.3% (997/1418) of the study participants reported ever had sexual partner in their life time. Of these women who had partners, 81.8% (816/997) reported that their partners were tested for HIV, and 73.8% (602/816) are positive for HIV. Of the total study participants, 463(32.7%) women reported having one child, 321(22.6%) two children and 428(30.2%) no children. About 59% (507/859) and 41% (352/859) HAART users and HAART naïve women, respectively, had disclosed HIV status to partner/husband. Furthermore, 66.6%(664/997) women reported they had open discussion with partner/husband (Table 2).

**Table 2:** Reproductive and clinical characteristics of HIV positive women in Addis Ababa, Ethiopia (n = 1418)

**Reproductive and clinical history**	**HAART users (n = 770)**	**HAART naïves (n = 648)**	**Total**
	**No (%)**	**No (%)**	**No (%)**
**Nadir CD4 count**			
0-199 cells/mm^3^	466 (79.2)	97 (20.8)	563 (39.7)
200-349 cells/mm^3^	225 (45.9)	265 (54.1)	490 (34.6)
350+ cells/mm^3^	79 (21.6)	286 (78.4)	365 (25.7)
**Current CD4 count**			
0-199 cells/mm^3^	125 (64.4)	69 (35.6)	194 (13.7)
200-349 cells/mm^3^	223 (49)	232 (51)	455 (32.1)
350+ cells/mm^3^	422 (55)	347 (45)	769 (54.2)
WHO stage of Disease			
Stage I/II	363 (42)	500 (58)	863 (60.9)
Stage III/IV	407 (73.3)	148 (26.7)	555 (39.1)
**Presence of partner**			
Yes	560 (56.2)	437 (43.8)	997 (70.3)
No	210 (50)	211 (50)	421 (29.7)
**Partner tested (n = 997)**			
Yes	479 (59)	337 (41)	816 (81.8)
No	47 (48)	50 (52)	97 (9.7)
I do not know	34 (40)	50 (60)	84 (8.4)
**Test result of current partner (n = 816)**			
Positive	347 (58)	255 (42)	602 (73.8)
Negative	119 (60)	73 (40)	199 (24.4)
I do not know	13 (87)	2 (13)	15 (1.8)
**Child tested (n = 990)**			
Yes	377 (58)	273 (42)	650 (65.6)
No	175 (51)	165 (40)	340 (34.3)
**Disclose HIV status to anybody**			
Yes	722 (56.3)	558 (43.7)	1280 (90.3)
No	48 (34.7)	90 (65.3)	138 (9.7)
**Disclosed HIV status to husband (n = 997)**			
Yes	507 (59)	352 (41)	859 (86.2)
No	53 (38.4)	85 (61.6)	138 (13.8)
**Number of living children**			
0	218 (51)	210 (40)	428 (30.2)
1	260 (51)	203 (49)	463 (32.7)
2	180 (56)	141 (44)	321 (22.6)
3+	112 (54.3)	94 (45.7)	206 (14.5)
**Discussed with husband/Partner (n = 997)**			
Yes	384 (57.8)	280 (42.2)	664 (66.6)
No	176 (52.8)	157 (47.2)	333 (33.4)
Discussed with HCWs*			
Yes	553 (54.7)	457 (45.3)	1010 (71.2)
No	217 (52.8)	191 (47.2)	408 (28.8)

*HCWs: health care workers.

### Use and preference of contraceptive methods

Overall contraceptive use was 71% (75% (579/770) and 65% (422/648) among HAART users and 42% HAART- naïve women, respectively). Four hundred five (28.8%) reported did not use contraceptive methods six months prior to the data collection period while contraceptive status was not known for 9 (0.6%) women (Table 3). In general male condom is the most preferred contraceptive method 45.7% (468/1001) followed by injectables 30.5% (306/1001) (Table 3).

**Table 3:** Use and preference of contraceptive methods among HIV positive women in Addis Ababa, Ethiopia

**Type of contraceptives**	**HAART users (n = 770)**	**HAART Naives (n = 648)**	**Total**
	**No (%)**	**No (%)**	**No (%)**
**Contraceptive use**			
Yes	579 (75.2)	422 (65.1)	1001 (70.6)
No	186 (24.2)	222 (54.3)	408 (28.8)
Not known	5 (0.6)	4 (0.6)	9 (0.6)
**Method preference**			
Injectables (Depot)	146 (25.2)	160 (37.9)	306 (30.5)
Oral contraceptive pills (OCP) of available type	55 (9.4)	46 (11)	101 (10.0)
Intra uterine contraceptive device (IUCD)	16 (2.7)	12 (2.8)	28 (2.8)
Implant (Jaddelle or Implanon)	48 (8.2)	45 (10.5)	93 (9.2)
Consistent male condom use	310 (53.5)	158 (37.4)	468 (46.7)
Female condom	4 (0.7)	1 (0.2)	5 (0.5)
**Categories of contraceptives methods**			
Dual contraceptive methods (condom and hormonal methods)	98 (14.7)	114 (21.6)	212 (18)
Barrier Methods (male or female condom)	317 (47.5)	161 (30.6)	478 (40)
Hormonal methods (OCPs, Implant and Depot)	249 (37.5)	251 (39.9)	500 (42)

N.B. a woman may have reported the use of ≥ 1 method value will not be added to 100%.

### Reasons given in choosing contraceptive methods

**Figure 1:**
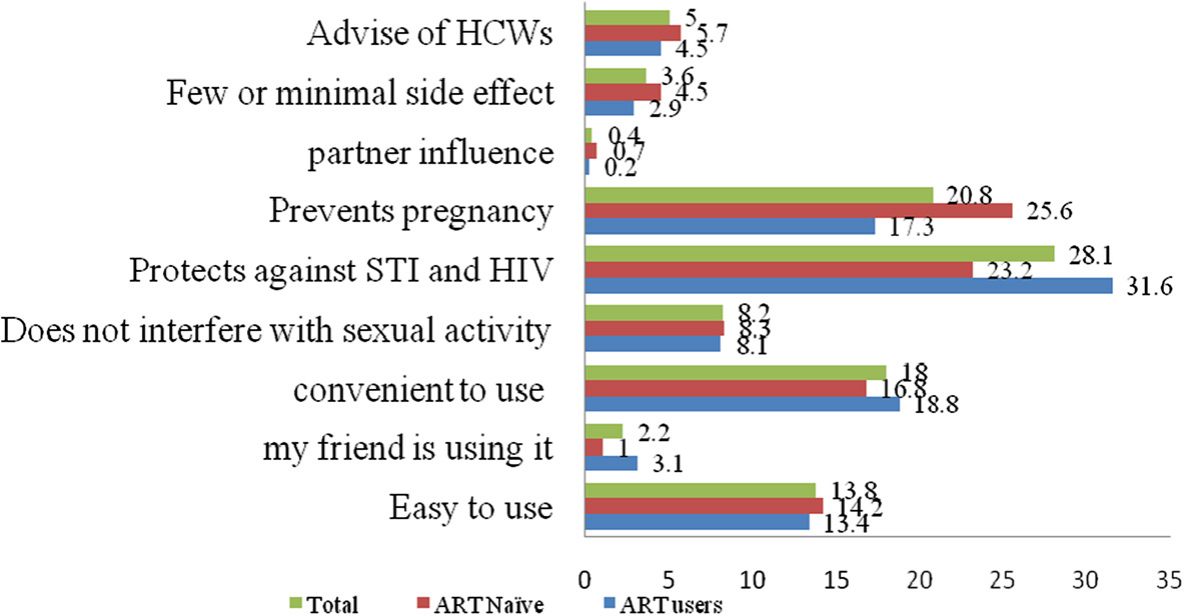
Reasons given in choosing methods of prevention among HIV positive women in Addis Ababa, Ethiopia.

The primary reason given for contraceptive use was for protection against STIs in 28% (281/1001) followed by prevention of unwanted pregnancy in 21% (208/1001), among women using any method. Other reasons mentioned were convenient to use 18% (180/1001), easy to use 14% (138/1001), does not interfere with sexual activity 8% (82/1001), advise of health workers 5% (50/1001), and the like (Figure 1).

### Factors associated with contraceptive use

Using the logistic regression model, a number of socio-demographic, HAART use, sexual history, disclosure behavior and clinical factors were identified as significant predictors of the association of contraceptive use (Table 4).

**Table 4:** Adjusted analyses of variables associated with contraceptive use among women in Addis Ababa, Ethiopia

**Variables**	***Contraceptive use***		***Adjusted odds ratio AOR (95% CI)***
	***Yes***	***No***	
	***No. (%)***	***No. (%)***	
**HAART use**			
No	422 (42.2)	226 (55.4)	1.00
Yes	579 (57.8)	191 (45.6)	1.6 (1.30-2.12)**
**Presence of partner**			
No	236 (26.6)	185 (44.4)	1.00
Yes	765 (76.4))	232 (55.6)	2.32 (1.60-3.40)**
**Disclosed HIV status to husband**			
No	87 (11.4)	51 (22.1)	1.00
Yes	679 (88.6)	180 (77.9)	2.23 (1.21-4.12)**
**Discussed with husband**			
No	240 (31.3)	99 (42.9)	1.00
Yes	526 (68.7)	132 (57.1)	2.0 (1.60-2.60)**
**Discussed with HWs**			
No	242 (24.9)	166 (39.8)	1.00
Yes	759 (75.1)	251 (60.2)	1.60 (1.20-2.03)**
**Number of living Children**			
0	275 (27.5)	153 (36.7)	1.00
1	329 (32.9)	134 (32.1)	1.7 (1.03-2.40)**
2	241 (24.1)	80 (19.2)	2.6 (1.7-4.02)**
3+	156 (15.6)	50 (12.0)	3.3 (1.90-5.60)**
**Marital status**			
Single	168 (16.8)	74 (17.7)	1.00
Married/cohabited	673 (67.2)	212 (50.8)	0.41 (0.21-0.80)*
Widowed	73 (7.3)	63 (15.1)	0.31 (0.12-0.84)*
Divorced	87 (8.7)	68 (16.3)	0.80 (0.30-2.30)
**Child tested for HIV**			
No	236 (32.5)	104 (39.3)	1.00
Yes	490 (67.4)	160 (60.6)	1.60 (1.10-2.40)*

*P value < 0.05 **P-value < 0.001.

Adjusted for age, education and other SES.

The results showed that HAART use has influence on women’s decision to use contraceptives compared to HAART naïve women, the odds of contraceptive use among HAART users was significantly higher, adjusted for other potential confounders (AOR 1.60, 95% CI; 1.30 to 2.12). Similarly, women who had sexual partners currently were more likely to use contraceptives than women with no partners (AOR 2.32, 95% CI 1.60 to 3.40). On the other hand, married/cohabited (AOR 0.41, 95% CI 0.21 to 0.80) and widowed women (AOR 0.31, 95% CI 0.12 to 0.84) respectively, had less likely to use contraceptive methods compared to that of single women.

Disclosure of HIV status, especially, with sexual partner has significant association with contraceptive use, women who disclosed their HIV status to partner were about two times more likely to use contraceptive than women who did not (AOR 2.23, 95% CI 1.21 to 4.12). Furthermore, women who reported open discussion with their partner/husband were about two times more likely to use contraceptives than those who reported no discussion (AOR 2.0, 95% CI; 1.60 to 2.60).

Contraceptive use also increased by the number of living children women have. The odds of contraceptive use for women who have one child (AOR 1.7, 95% CI 1.03 to 2.40), two children (AOR 2.6; 95% CI 1.7 to 4.02) and three or more children (AOR 3.3, 95% CI 1.90 to 5.60), respectively, were higher than women with no children. Similarly, the odds of contraceptive use was higher for women whose children are tested for HIV than women who did not (AOR, 1.6; 95% CI; 1.01 to 2.40).

## Discussion

The issue of contraceptive use and method preference among women enrolled in HIV care and treatment programs in the study area has important implications for the health of women and their infants. Overall the proportion of contraceptive use was 71% irrespective of their HAART use. Previous studies from Uganda and South Africa reported higher than our finding 85% and 78%, respectively [5,12]. On the other hand, our finding showed higher proportion than previous findings from other developing countries, which reported between 28 and 53% [39-42] and than earlier reports within the country reported 43 to 54% [29,30,43]. The difference is associated with study time and study subject differences.

The result is different from contraceptive use of the general female population in Addis Ababa reported 63% [44]. The most probable reason might be that HIV positive women have frequent contact with health care providers and demand for contraceptives might be higher than the general population.

In the logistic model, we found that, adjusted for potential confounders, contraceptive use was significantly higher among women receiving HAART than HAART naïve; which was also reported by previous studies from Uganda and South Africa [5,12]. This may be a reflection of the fact that continual exposures to secondary prevention messages along with ART might have impact on contraceptive use. Even though, male condom is the most preferred method by all study participants, it was higher among those receiving HAART compared to HAART naïve women (54 vs 37%, respectively).

While the use of dual contraceptive methods (condom with other methods) is safe to prevent unintended pregnancy and HIV [25,45]. However, low proportion (14.7%) of dual contraceptive method use was observed among HAART users. This may reflect that the study population is at risk of acquiring sexually transmitted infection including drug resistant HIV virus.

Our findings has also showed that women with living children have reported contraceptive use than those with no children; the result was consistent with studies reported from Uganda and South Africa [5,12]. This suggests that the desire to have children among those with no children is higher for obvious reasons and consequently avoiding contraceptive use.

Furthermore, women who have open discussion with partner or health care providers have better contraceptive profile than their counterparts. This suggests that disclosure of HIV status to a partner may be important to get support from family and discussion can clarify uncertainties about contraceptives and possibly to strengthen confidence of women. Besides women who knew the HIV status of their children reported contraceptive use than their counter parts, this could be related to women who tested their children have better exposure and more concerned on the prevention of unintended pregnancies.

Our study has several limitations; due to cross-sectional nature we are unable to make definitive conclusions on cause and effect relation. The association could only be discussed in terms of plausibility. Furthermore, social desirability and stigma may have biased respondents’ answers and may not be generalizable to HIV-positive female population in the care.

As to the strengths of this study, the respondents have been selected by random sampling technique with relatively large sample size. Again, the team already adopted instrument conducted in other developing countries [12]. Precautions have been taken in selection of experienced data collectors.

## Conclusion

The study identified that HIV positive women in general and women receiving HAART in particular are more likely to use contraceptives with preference of male condom. Its protection against STIs followed by prevention of unwanted pregnancy and convenient to use were among the reasons mentioned for selection of the contraceptive methods. Furthermore, having one or more living children, disclosure of HIV status to sexual partner, open discussion with partner or health care workers found to be predictors of contraceptive use in the study area. Low proportion of dual contraceptive method use was observed among HAART users. This may reflect that the study population is at risk of acquiring sexually transmitted infection including drug resistant HIV virus.

The contraceptive profile of women in the study area mainly dependent on male condom use, this indicates the need to better integrate tailored counseling and contraceptive options with care and support activities that targets HIV positive women. Moreover, emphasis should be given to dual contraceptive method use along with their regular follow up irrespective of their HAART use.

Lastly extensive and longitudinal study is needed to validate current findings so as to inform for policy makers to establish better sexual and reproductive health services for positive women to have planed and safe fertility goal.

## Competing interests

We declare that there are no financial or non-financial competing interests related to this study.

## Authors’ contributions

Both authors contributed equally during design and conduct of the study. HM and FE participated in data collection, statistical analysis and interpretation of findings. HM prepared the draft then FE revised the draft of the paper. The two authors read and approved the final content of the manuscript.

## Pre-publication history

The pre-publication history for this paper can be accessed here:

http://www.biomedcentral.com/1471-2458/14/566/prepub

## References

[B1] UNAIDS WHO Global HIV/AIDS Response-Epidemic update and health sector progress towards Universal Access-Progress Report 2011 Geneva, Switzerland: UNAIDS

[B2] UNAIDS Global Report, “UNAIDS report on Global AIDS Epidemic,” 2012 Geneva,Switzerland: UNAIDS

[B3] UNAIDS Report on HIV Epidemic in Eastern and Southern Africa 2013 Geneva, Switzerland: Regional Report UNAIDS

[B4] Federal Ministry of Health Ethiopia FMoHE Health and Health Related Indicators 2011 Addis Ababa, Ethiopia: FMOHE

[B5] Andia I Kaida A Maier M Guzman D Emenyonu N Pepper L David R Robert S Highly active antiretroviral therapy and increased use of contraceptives among HIVpositive women during expanding access to antiretroviral therapy in Mbarara, Uganda Am J Public Health 2009 99 2 340 347 10.2105/AJPH.2007.129528 19059862 PMC2622797

[B6] Koenig LJ Espinoza L Hodge K Ruffo N Young, sero positive, and pregnant: epidemiologic and psychosocial perspectives on pregnant adolescents with human immunodeficiency virus infection Am J Obstet Gynecol 2007 197 3 Supple S123 S131 17825643 10.1016/j.ajog.2007.03.004

[B7] Zimbwa I Vwalika B Association Between Unplanned Pregnancy and HIV Seropositivity Disclosure to Marital/Cohabitating Partner Among Post-natal Women in Lusaka, Zambia Med J Zambia 2010 37 4 205 215

[B8] Hubacher D Mavranezouli I McGinn E Unintended pregnancy in sub-Saharan Africa: magnitude of the problem and potential role of contraceptive implants to alleviate it Contraception 2008 78 73 78 10.1016/j.contraception.2008.03.002 18555821

[B9] Schwartz SR Rees H Mehta S Venter WDF Taha TE Black V High Incidence of Unplanned Pregnancy after Antiretroviral Therapy Initiation: Findings from a Prospective Cohort Study in South Africa PLoS ONE 2012 7 4 e36039 10.1371/journal.pone.0036039 22558319 PMC3338622

[B10] Akelo V Girde S Borkowf CB Angira F Achola K Lando R Mills LA Thomas TK Shirley Lee L Attitudes toward Family Planning among HIV-Positive Pregnant Women Enrolled in a Prevention of Mother-To-Child Transmission Study in Kisumu, Kenya PLOS ONE 2013 8 8 e66593 doi:10.1371/journal.pone.0066593 10.1371/journal.pone.0066593 23990868 PMC3753279

[B11] Federal Ministry of Health Ethiopia FMoHE, Federal HIV/AIDS Prevention and Control Office FHAPCO Guidelines for Prevention of Mother-to-Child Transmission of HIV in Ethiopia 2007 Addis Ababa: FMoH 1 3

[B12] Kaida A Laher F Strathdee SA Money D Janssen PA Hogg R Gray G Contraceptive Use and Method Preference among Women in Soweto South Africa The Influence of Expanding Access to HIV Care and Treatment Services PLoS ONE 2010 5 11 e13868 doi:10.1371/journal.pone.0013868 10.1371/journal.pone.0013868 21079770 PMC2974641

[B13] Raynols HW Janowitiz B Homan R Johonson L The value of contraception to Prevent Perinatal HIV Transmission Sex Transm Dis 2006 33 6 350 356 10.1097/01.olq.0000194602.01058.e1 16505747

[B14] World Health Organization WHO Communicating Family Planning in Reproductive Health Geneva, Switzerland: WHO Available from: http://www.who.int/entity/reproductivehealth/publications/family_planning

[B15] Baylies C The impact of HIV on family size preference in Zambia Reprod Health Matters 2000 8 77 86 11424271 10.1016/s0968-8080(00)90008-9

[B16] USAID Priorities for Family Planning and HIV/AIDS Integration, Maximizing Access and Quality (MAQ) Initiativ Available from: http://www.maqweb.org

[B17] Cleland J Bernstein S Ezeh A Faundes A Glasier A Innis J Family Planning the unfinished agenda Lancet 2006 368 1810 27 10.1016/S0140-6736(06)69480-4 17113431

[B18] UNFPA Preventing HIV and Unintended Pregnancies Strategic Framework 2011–2015. Available from: http://www.unfpa.org/public/home/publications/pid/10575.

[B19] 19.World Health Organization PMTCT Strategic Vision 2010–2015 2010 WHO Geneva, Switzerland: Preventing Mother-to-Child Transmission of HIV to Reach the UNGASS and Millennium Development Goals

[B20] Petruney T Robinson E Reynolds H Wilcher R Cates W Contraception is the best kept secret for prevention of mother-to-child HIV transmission 2008 Bulletin World Health Organ 86 B. doi:10.2471/BLT.08.051458 available: http://www.ncbi.nlm.nih.gov/pmc/articles/PMC2647467/10.2471/BLT.08.051458 PMC2647467 18568260

[B21] Ngom P Wilcher R Kuyoh M Dube H Martin S Kimani J Nutley T Maggwa N Family planning needs in the context of the HIV/AIDS epidemic Findings from a three-country assessment 2005 Kenya, South Africa and Zimbabwe: XXV IUSSP International Conference Tours; France

[B22] Stefiszyn K Health and Reproductive rights, HIV and the protocol to the African Charter on the right of women in Africa ESR Review 2011 12 4 12 15

[B23] World Health Organization Use of antiretroviral drugs for treating pregnant women and preventing HIV infection in infants 2012 Geneva, Switzerland: WHO Available from: http://whqlibdoc.who.int/hq/2012/who_hiv_2012.8_eng.Pdf 26180894

[B24] Iliyasu Z Abubakar I Kabir M Babashani M Shuaib F Aliy MH Correlates of Fertility Intentions among HIV/AIDS Patients in Northern Nigeria Afr J Reprod Health 2009 13 3 71 83 20690263

[B25] Mitchell HS Stephens E Contraception choice for HIV positive women Sex Transm Infect 2004 80 3 163 77 10.1136/sti.2003.008441 PMC1744846 15169996

[B26] Heard I Potard V Costagliola D Kazatchkine MD Contraceptive use in HIV-positive women J Acquir Immune Defic Syndr 2004 36 714 20 10.1097/00126334-200406010-00008 15167290

[B27] Massad LS Evans CT Wilson TE Golub ET Sanchez-Keeland L Minkoff H Weber K Watts DH Contraceptive use among U.S.women with HIV J Women’s Health 2007 5 657 666 10.1089/jwh.2006.0204 17627401

[B28] Debeko K Seme A Sexual and reproductive health needs and preferences of people living with HIV/AIDS in Southern Nations Nationalities and Peoples Region 2007 Addis Ababa University, Department of Community Health: Masters thesis unpublished

[B29] Worku D Fertility intension and Demand for Family Planning among People on ART Follow up in North Wollo 2010 Ethiopia: 21ST Annual Public Health Conference 12

[B30] Tamene W Fantahun M Fertility desire and family-planning demand among HIV-positive women and men undergoing antiretroviral treatment in Addis Ababa, Ethiopia Afr J AIDS Res 2007 6 3 223 7 10.2989/16085900709490418 25866168

[B31] Federal Ministry of Health Ethiopia FMoHE National Comprehensive PMTCT/MNCH Training Package, Facilitator’s Guide 2013 Addis Ababa, Ethiopia: FMoHE 11 25

[B32] Getachew M Alemseged F Abera M Deribew A Factors affecting fertility decisions of married men and women living with HIV in South Wollo Zone North east Ethiopia Ethiop J Health Dev 2010 24 3 214 20

[B33] Federal Ministry of Health Ethiopia FMOH Antiretroviral Therapy Guidelines for Adult Patients in Ethiopia 2008 Addis Ababa,Ethiopia: FMOHE

[B34] Federal Ministry of Health Ethiopia FMOHE, Federal HIV/AIDS Prevention and Control Office FHAPCO Monthly ART Update as of end of Tir 2002 E.C 2010 Addis Ababa,Ethiopia Available from: http://www.hapco.gov.et/index.php/resource-center/art-monthly-updates

[B35] Central Statistics Agency of Ethiopia CSA, ORC Macro USA Census Result 2007 2007 Addis Ababa, Ethiopia: CSA 11

[B36] Barthlett JG, Gallant JE Medical management of HIV Infection: Ordering Information 2007 2 Baltimore, USA: John Hopkins University School of Medicine, John Hopkins 24 25

[B37] Federal Ministry of Health FMoHE, I-TECH-Ethiopia Comprehensive ART Training Manual for Physicians, Pharmacists and Nurses FMoHE 2007 1 1 10 12

[B38] Federal Ministry of Heaalth FMoHE Guideline for Adult HIV/AIDS care and treatment in Ethiopia 2008 2 Addis Ababa: FMoHE 6 7

[B39] Nóbreg AA Oliveira FA Galvão MT Mota RS Barbosa RM Dourado I Kendall C Kerr-Pontes LR Desire for a Child Among Women Living with HIV/AIDS in Northeast Brazil AIDS Patient Care STDs 2007 21 4 261 267 10.1089/apc.2006.0116 17461721

[B40] Muyindike W Fatch R Steinfield R Matthews LT Musinguzi N Emenyonu NI Martin JN Hahn JA Contraceptive Use and Associated Factors among Women Enrolling into HIV Care in Southwestern Uganda Infect Dis Obstet Gynecol 2012 2012 1 9 http://dx.doi.org/10.1155/2012/340782 10.1155/2012/340782 PMC3469089 23082069

[B41] Ezechi OC Gbajabiamilla TA Gab-Okafor CV Oladele DA Ezeobi PM O.Ujah IA Contraceptive Behavior, Practices and associated factors among Nigerian women Livingwith Human Immunodeficiency virus infection J HIV& Hum Reprod 2013 1 1 30 35 10.4103/2321-9157.116528

[B42] Umoh AV Abah GM Ekanem US A study of fertility intentions of women in Uyo, Nigeria J Public Health Epidemiol 2012 4 1 14 8

[B43] Assefa B Fertility desire and FP use in PLWH on pre-ART and ART care in public facilities of Addis Ababa City Administration EPHA Sponsored Masters thesis 2009 12 12 131 136

[B44] Central Statistics Agency,CSA Ethiopia Ethiopia Demographic and Health Survey Report 2011 Addis Ababa, Ethiopia: CSA

[B45] World Health Organization, UNFPA Sexual and reproductive health of women living with HIV/AIDS Guidelines on care, treatment and support for women living with HIV/AIDS and their children in resource-constrained settings 2006 30 35

